# Macrophages exploit the mannose receptor and JAK-STAT1-MHC-II pathway to drive antigen presentation and the antimycobacterial immune response after BCG vaccination: MR deficiency impairs BCG immune response

**DOI:** 10.3724/abbs.2024100

**Published:** 2024-06-18

**Authors:** Ying Zhang, Dandan Xu, Qi Nie, Jing Wang, Dan Fang, Yan Xie, Huang Xiong, Qin Pan, Xiao-Lian Zhang

**Affiliations:** 1 Department of Immunology Wuhan University Taikang Medical School (School of Basic Medical Sciences) Department of Allergy of Zhongnan Hospital and Hubei Province Key Laboratory of Allergy and Immunology Wuhan University Wuhan 430071 China; 2 State Key Laboratory of Virology Medical Research Institute and Frontier Science Center for Immunology and Metabolism Wuhan University Wuhan 430071 China; 3 Department of Blood Transfusion the Affiliated Xuzhou Municipal Hospital of Xuzhou Medical University Xuzhou First People’s Hospital Xuzhou 221116 China; 4 Wuhan Jinyintan Hospital Tongji Medical College of Huazhong University of Science and Technology Wuhan 430023 China; 5 Department of Anatomy Wuhan University Taikang Medical School (School of Basic Medical Sciences) and Hubei Province Key Laboratory of Allergy and Immunology Wuhan 430071 China

**Keywords:** BCG vaccine, mannose receptor, macrophage, antigen presentation

## Abstract

Tuberculosis (TB), caused by *Mycobacterium tuberculosis* ( *M*. *tb*), remains one of the leading causes of fatal infectious diseases worldwide. The only licensed vaccine, *Mycobacterium bovis* Bacillus Calmette-Guérin (BCG), has variable efficacy against TB in adults. Insufficiency of immune cell function diminishes the protective effects of the BCG vaccine. It is critical to clarify the mechanism underlying the antimycobacterial immune response during BCG vaccination. Macrophage mannose receptor (MR) is important for enhancing the uptake and processing of glycoconjugated antigens from pathogens for presentation to T cells, but the roles of macrophage MR in the BCG-induced immune response against *M*. *tb* are not yet clear. Here, we discover that macrophage MR deficiency impairs the antimycobacterial immune response in BCG-vaccinated mice. Mechanistically, macrophage MR triggers JAK-STAT1 signaling, which promotes antigen presentation via upregulated MHC-II and induces IL-12 production by macrophages, contributing to CD4 ^+^ T cell activation and IFN-γ production. MR deficiency in macrophages reduces the vaccine efficacy of BCG and increases susceptibility to *M*. *tb* H37Ra challenge in mice. Our results suggest that MR is critical for macrophage antigen presentation and the antimycobacterial immune response to BCG vaccination and offer valuable guidance for the preventive strategy of BCG immunization.

## Introduction

Tuberculosis (TB), caused by *Mycobacterium tuberculosis* ( *M*. *tb*), is one of the top 10 causes of death worldwide. According to a report by the World Health Organization, there were 10.6 million new cases of active TB and almost 1.3 million deaths from TB infections in 2022 [1]. The only licensed vaccine against TB, *Mycobacterium bovis* Bacillus Calmette-Guérin (BCG), mainly induces an IFN-γ-producing T helper type 1 (Th1) CD4 ^+^ T cell response and is effective in preventing TB among infants and children. However, the vaccine fails to confer sufficient protection against TB in adults [ 2, 3]. Insufficient immune cell function diminishes the protective effects of the BCG vaccine. It is critical to clarify the mechanism underlying the antimycobacterial immune response during BCG vaccination.

Macrophages are crucial immune cells during the antimycobacterial immune response. Macrophages utilize pattern recognition receptors (PRRs) to directly identify a variety of pathogen-associated molecular patterns (PAMPs) of *M*. *tb*, capture bacteria, and ingest them into the phagosome. The engulfed *M*. *tb* are lysed, and bacterial proteins are degraded by proteinases in the phagolysosome, which is formed by the fusion of the phagosome and lysosome within macrophages [4]. Upon *M*. *tb* infection, macrophages also produce IL-12 and reactive oxygen species (ROS), a group of reactive molecules derived from molecular oxygen, which have direct and indirect antimicrobial immune activity [5]. Although macrophage-induced robust innate immune responses are necessary for the early clearance of *M*. *tb* infection, these cells also function as antigen-presenting cells (APCs) and present processed *M*. *tb* antigens via major histocompatibility complex (MHC) molecules for T cell activation [6].

*M*. *tb* has unique molecular patterns, including lipoprotein and lipoglycan on the bacterial envelope, which are recognized by macrophage PRRs. The macrophage mannose receptor (MR, also termed CD206) plays a central role in enhancing the uptake and processing of glycoconjugated antigens from pathogens for presentation to T cells, but the roles of macrophage MR in the BCG-induced immune response against *M*. *tb* are not yet clear. MR, a PRR, belongs to the C-type lectin family and is highly expressed on not only macrophages but also myeloid dendritic cells (DCs) [7]. The receptor consists of an N-terminal cysteine-rich (CR) domain, a fibronectin (FN) type II domain, eight C-type carbohydrate recognition domains (CRDs), a transmembrane region and a short cytosolic region [8]. MR CRDs recognize mannosylated lipoprotein and lipoglycan of *M*. *tb* and regulate endocytosis, phagocytosis, and immune responses during bacterial infection [9].

In the present study, our data showed that MR is critical for macrophage antigen uptake and presentation via the p-JAK-p-STAT1-MHC-II axis for CD4 ^+^ T-cell activation in the BCG vaccine-induced antimycobacterial immune response. Our findings, based on the host MR, provide valuable insight into the mechanism of MR-involved engulfment and antigen presentation for BCG immunization.

## Materials and Methods

### Animals

The *Mr* ^‒/‒^ mice (C57BL/6J genetic background) were custom generated by the Animal Experiment Center of Wuhan University, Institute of Model Animal, Wuhan University. Wild-type (WT) C57BL/6J mice were procured from the same institution’s Experimental Animal Center. All mice utilized in the study were housed within specific pathogen-free (SPF) facilities at the Animal Experiment Center of Wuhan University. The animal experiments herein were conducted in accordance with the Guidelines of the China Animal Welfare Legislation and approved by the Committee on Ethics in the Care and Use of Laboratory Animals of Wuhan University (SQ20200031, WP20220095).

### Cell culture and stimulation

Murine peritoneal macrophages were harvested from the peritoneal lavage fluid of mice. The cells were cultured in Dulbecco’s modified Eagle’s medium (DMEM; Gibco, Carlsbad, USA) supplemented with 10% fetal bovine serum (FBS; Gibco) and laid on a cell culture dish. After 24 h of culture, the adherent cells were peritoneal macrophages.

Murine BMDMs were prepared from bone marrow cells by incubation in DMEM supplemented with 10% FBS plus macrophage colony-stimulating factor (M-CSF; 40 ng/mL; PeproTech, Suzhou, China) for 7 days.

### Bacterial culture

BCG (Pasteur strain ATCC 35734) and *M*. *tb* H37Ra (ATCC 25177) were propagated from laboratory stocks (Wuhan University Taikang Medical School, Wuhan, China). The mycobacterial strains were grown in Middlebrook 7H9 broth (BD Biosciences, Franklin Lakes, USA) supplemented with 10% oleic acid-albumin-dextrose-catalase (OADC; BD Biosciences) and 0.05% Tween 80 (Sigma-Aldrich, St Louis, USA) or on Middlebrook 7H10 agar (BD Biosciences) supplemented with 10% OADC.

### Magnetic activated cell sorting (MACS)

CD3 ^+^ T cells were purified and isolated from murine splenocytes using a negative control CD3 ^+^ T Cell Isolation Kit (#130-094-973; Miltenyi Biotec, Bergisch Gladbach, Germany).

### *In vitro* cell stimulation

For the detection of engulfed fluorescent BCG and microparticles, WT and *Mr* ^‒/‒^ BMDMs were stimulated with BCG (MOI=10) or microparticles (cell:microparticle=1:10) for 1 h. The cells were collected and washed with PBS, and the engulfed fluorescent BCG and microparticles were analyzed by FCM and confocal fluorescence microscopy.

To detect surface protein expression and cytokine production in macrophages upon BCG stimulation, WT and *Mr* ^‒/‒^ BMDMs were stimulated with BCG (MOI=10) for 24 h or treated with the STAT1 inhibitor fludarabine (1 μM) for 72 h prior to BCG stimulation.

For mixed lymphocyte reaction (MLR) analysis, WT and *Mr* ^‒/‒^ BMDMs were incubated with iBCG (MOI=10) for 24 h. After removing the extracellular iBCG by washing, the BMDMs were mixed with MACS-isolated CD3 ^+^ T cells in the presence/absence of anti-IL-12 (0.1 μg/mL; Bioxcell, Shanghai, China) for 3 days. The expression of antigen-presenting genes on BMDMs, cytokine production by BMDMs and T cells, and T cell proliferation were measured by FCM.

### Flow cytometry (FCM)

For the detection of cell surface phenotypes, cells were stained with APC-cy7-FVS780, APC-anti-mouse-F4/80 (QA17A29), APC-anti-mouse-CD3 (145-2C11), PC7-anti-mouse-CD4 (GK1.5), PE-anti-mouse-CD80 (16-10A1), PC-anti-mouse-CD86 (145-2C11), APC-anti-mouse-MHC I (695H1-9-9), and PE-anti-mouse-MHC II (M1/42) antibodies. After incubation for 30 min at 4°C in the dark, the labelled cells were washed twice before being analyzed by FCM. To analyze T-cell proliferation, CD4 ^+^ T cells were labelled with 1.5 μM CFSE dye prior to being mixed with BMDMs.

For the detection of intracellular molecules, BMDMs and CD4 ^+^ T cells were fixed/permeabilized with Cytofix/Cytoperm (BD Biosciences), followed by antibody staining. The antibodies used were PE-conjugated anti-mouse IL-12 (554479), PE-conjugated anti-mouse IL-4 (11B11), PE-conjugated anti-mouse IL-10 (JES5-16E3), PE-conjugated anti-mouse IL-2 (554429), PE-conjugated anti-mouse IFN-γ (XMG1.2), and PE-conjugated anti-mouse TNF-α (MP6-XT22). To detect ROS production by macrophages, the cells were incubated with 10 μM dichlorodihydrofluorescein diacetate (DCFH-DA) in serum-free cell culture medium for 20 min at 37°C in the dark.

All the antibodies were purchased from BD Biosciences and BioLegend. The cells were analyzed with a FACSCanto™ II, FACSAria™ III flow cytometer (BD Biosciences) or Beckman CytoFLEx (Beckman, Brea, USA) at the Medical Structural Biology Research Center of Wuhan University.

### Confocal microscopy analysis

BMDMs were incubated with BCG (MOI=10) or microparticles (cell: microparticle=1:10) for 1 h. Extracellular BCG and microparticles were removed by washing the cells with phosphate-buffered saline (PBS) three times. The cells were fixed with 4% paraformaldehyde for 10 min, further cultured for 2 h and stained with an anti-mouse STAT1 antibody (A19563; ABclonal, Wuhan, China) at 4°C overnight. Alternatively, after 24 h of further incubation, the cells were fixed and stained with anti-mouse/human ATP6V1A (17115-1-AP; Proteintech, Chicago, USA). After washing, the cells were stained with DAPI for 2-5 min prior to confocal microscopy analysis.

To detect the acidity of macrophage phagosomes, BMDMs were stimulated with BCG for 4 h. After the cells were washed with PBS, they were suspended in serum-free cell culture medium and incubated with 1 μM LysoSensor Yellow/Blue DND-160 (Yeasen Biotechnology Co., Ltd., Shanghai, China) for 30 min at 37°C in the dark.

### Immunoblotting analysis

WT and *Mr* ^‒/‒^ BMDMs were stimulated with BCG for 0.5, 1 and 3 h. The phosphorylation of JAK and STAT1 in the cytoplasm and nucleus of the cells was measured. Nuclear and cytoplasmic proteins were extracted using a Nuclear and Cytoplasmic Protein Extraction kit (Beyotime Biotechnology, Shanghai, China). The proteins were quantified using a bicinchoninic acid (BCA) protein quantification kit (Servicebio, Shanghai, China), separated on an SDS-polyacrylamide gel, and transferred onto polyvinylidene difluoride membranes (Millipore, Billerica, USA). Specific primary antibodies and HRP-conjugated secondary antibodies were used to identify particular antigens. Bound antibodies were detected with and visualized by chemiluminescence (Dalian Meilun Biology Biotechnology Co., Ltd., Dalian, China). The antibodies used were as follows: anti-human/mouseβ-actin (2D4H5; Proteintech), anti-rabbit/mouse STAT1 (ab92506; Abcam, Cambridge, UK), anti-mouse phospho-STAT1 (Ser727; ab109461; Abcam), anti-mouse JAK1 (AF5012; Affinity, Houston, USA) and anti-mouse phospho-JAK1 (Tyr1022/Tyr1023/Tyr1034/Tyr1035; AF2012; Affinity).

### RNA-seq and sample preparation

WT and *Mr* ^‒/‒^ BMDMs were stimulated with BCG for 1 h. After removing the extracellular BCG by washing with PBS three times and further culturing for 11 h, the cell pellets were collected and resuspended in Trizol reagent (Invitrogen, Carlsbad, USA). The samples were then sent to Novogene Company (Beijing, China) for RNA sequencing and analysis.

### Reverse transcription quantitative PCR (RT-qPCR)

RT-qPCR was used to perform quantitative analysis of RNA molecules. WT and *Mr* ^‒/‒^ BMDMs were stimulated with BCG for 6 h. Total RNA from the BMDMs was isolated with Trizol reagent (Invitrogen). cDNA synthesis and real-time PCR were performed using RT Master Mix (Toyobo, Tokyo, Japan) and SYBR Green real-time PCR mix (Toyobo) with specific primers ( Supplementary Table S1). Target gene expression levels were normalized to those of glyceraldehyde-3-phosphate dehydrogenase ( *GAPDH*). Relative RNA levels were calculated by the comparative cycle threshold (Ct) method (2 ^−ΔΔCt^ method), where Ct indicates the amplification cycle number at which the fluorescence generated within a reaction rises above a defined threshold fluorescence.

### Bacterial colony counting assay

WT and *Mr* ^‒/‒^ BMDMs were treated with BCG (MOI=10) for 1 h and washed with PBS three times. The cells were then incubated in gentamicin (200 μg/mL) for 0.5 h to eliminate the extracellular BCG. After washing, the cells were further cultured for 2, 12, or 24 h. The BMDMs were lysed in PBS supplemented with 0.2% Triton X-100 for 5 min. The cell lysates were plated on 7H10 medium. After 21 days of culture, the bacterial colonies were counted.

### Murine model of BCG vaccination and *M*. *tb* H37Ra infection

The subcutaneous ( *s*. *c*.) vaccination of BCG confers dose-dependent protection [ 10– 12]. To boost the immune recall response, doses of 10 ^7^‒10 ^8^ CFUs are commonly used for BCG in murine models [ 10– 12]. In the BCG vaccination model, WT and *Mr* ^‒/‒^ mice were subcutaneously vaccinated with BCG (1×10 ^8^ BCG/mouse) on day 0. On day 30, the splenocytes of the mice were harvested. The surface proteins of antigen-presenting-related molecules and cytokine production were measured by FCM. To detect the BCG-specific CD4 ^+^ T-cell response, splenocytes from the mice were restimulated with the mycobacterial antigen Ag85B peptide P25 (10 μg/mL, NH2-FQDAYNAAGGHNAVF-COOH) and iBCG (MOI=10) for 72 h before cytokine production was measured by FCM analysis.

In the murine model of *M*. *tb* H37Ra infection, WT and *Mr* ^–/–^ mice were vaccinated with BCG on day 0, and the mice were nasally infected with *M*. *tb* H37Ra on day 30. After 7 days of infection, the bacterial burden and pathological changes in the lungs were measured.

### Hematoxylin and eosin (H&E) staining

Mouse lungs were inflated and fixed in 10% neutral buffered formalin, processed and embedded in paraffin, sectioned at 5 μm and stained with H&E. The stained glass slides were magnified 10 times and digitally scanned by Leica Aperio VERSA 8 (Leica, Wetzlar, Germany) at 0.23 microns per pixel. The median image sizes are 1148× 631 pixels.

### Statistical analysis

FlowJo_V10 software (BD Biosciences) was used for flow cytometry analysis. GraphPad Prism software 8.0.1 (GraphPad Software, Inc., San Diego, USA) was used for the statistical analyses. Statistical differences between groups were analyzed using two-tailed unpaired Student’s *t* test or one-way ANOVA followed by Newman-Keuls post hoc test. Data are presented as the mean±standard deviation (SD). *P*<0.05 was considered statistically significant.

## Results

### MR deficiency impairs phagocytosis of BCG by macrophages *in vitro*

**Figure 1:**
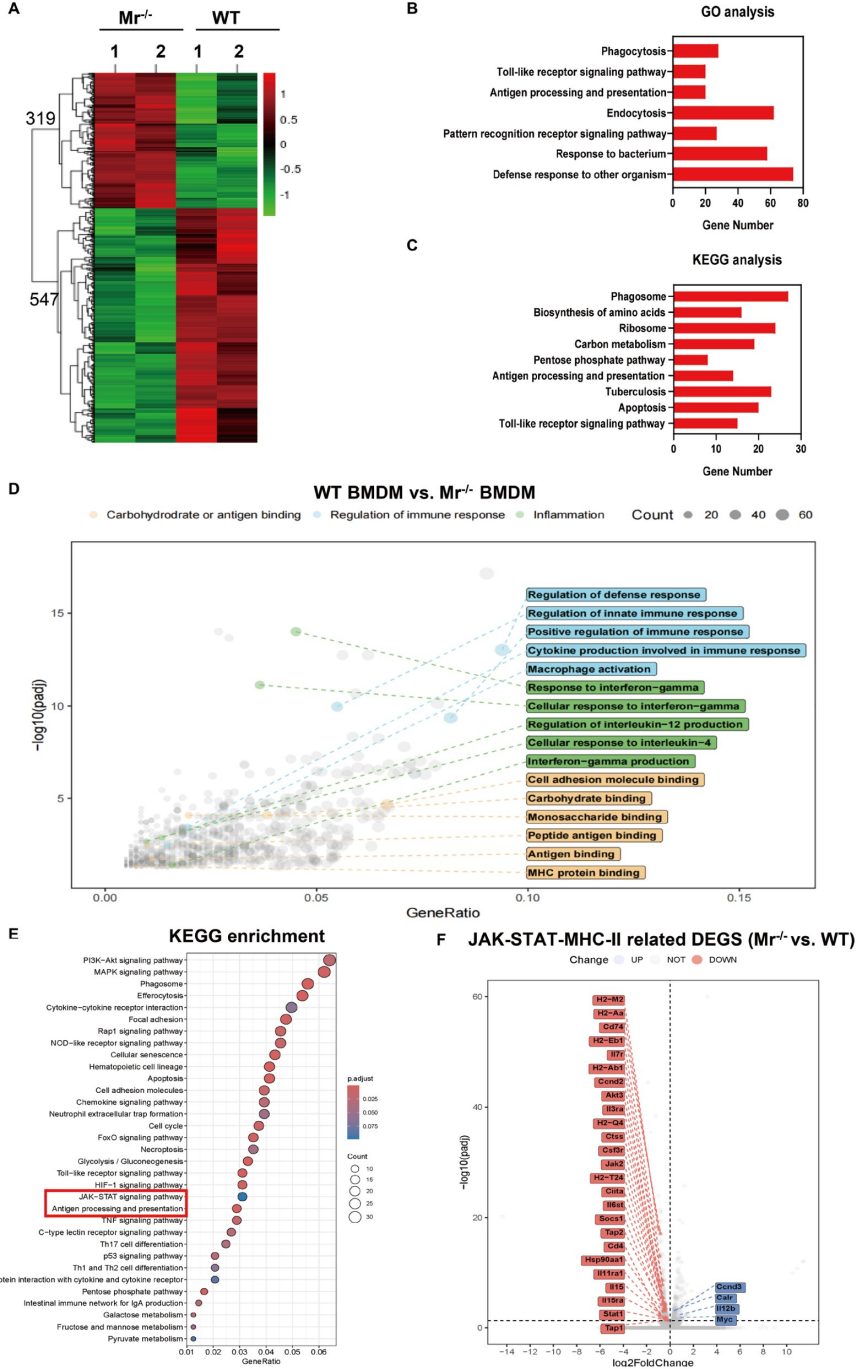
Figure 1 RNA-seq and functional annotation of *Mr* ^‒/‒^ BMDMs compared with WT BMDMs using GO and KEGG terms WT and Mr‒/‒ BMDMs were stimulated with BCG for 1 h. After the extracellular bacteria were removed by washing, the cells were further cultured for 11 h, and the total RNA of the cells was analyzed by RNA-seq. (A) Heatmap of differentially expressed genes. (B,C) Functional annotation using GO (B) and KEGG terms (C). (D) GO term analysis of differentially expressed genes. WT BMDMs vs Mr‒/‒ BMDMs. padj: adjusted p value. (E) KEGG pathway enrichment analysis. WT BMDMs vs Mr‒/‒ BMDMs. (F) Differentially expressed genes from RNA-seq analysis. JAK-STAT1-MHC-II pathway-related genes are highlighted.

To determine the role of MR in macrophages during BCG vaccination, we generated MR knockout ( *Mr* ^‒/‒^) mice and isolated bone marrow-derived macrophages (BMDMs) from WT or *Mr* ^‒/‒^ mice. We employed RNA sequencing (RNA-seq) to assess differential gene expression (DEGs) between WT and *Mr* ^‒/‒^ BMDMs incubated with BCG for 12 h *in vitro* ( Figure 1A). A total of 319 genes were significantly upregulated, and 547 genes were significantly downregulated in the *Mr* ^‒/‒^ group compared with the WT group ( Figure 1A). Most of the genes were associated with phagocytosis, the Toll-like receptor signaling pathway, antigen processing and presentation, endocytosis and the pattern recognition receptor signaling pathway according to the GO and KEGG analysis ( Figure 1B,C). Gene Ontology (GO) analysis revealed three groups of GO terms related to inflammation, carbohydrate or antigen binding and immune response regulation ( Figure 1D). Compared to those in the *Mr* ^‒/‒^ group, the pathways related to MHC protein binding and IL-12 production signaling were more enriched in the WT group. Additionally, KEGG analysis revealed that the “JAK-STAT signaling pathway”, “antigen presentation” and “cytokine‒cytokine receptor interaction” pathways were enriched in the WT group ( Figure 1E). DEC analysis revealed that several genes related to the JAK-STAT1-MHC-II pathway were upregulated in the WT group ( Figure 1F). These results reveal that MR is highly related to phagocytosis, antigen uptake, processing and presentation by macrophages.

**Figure 2:**
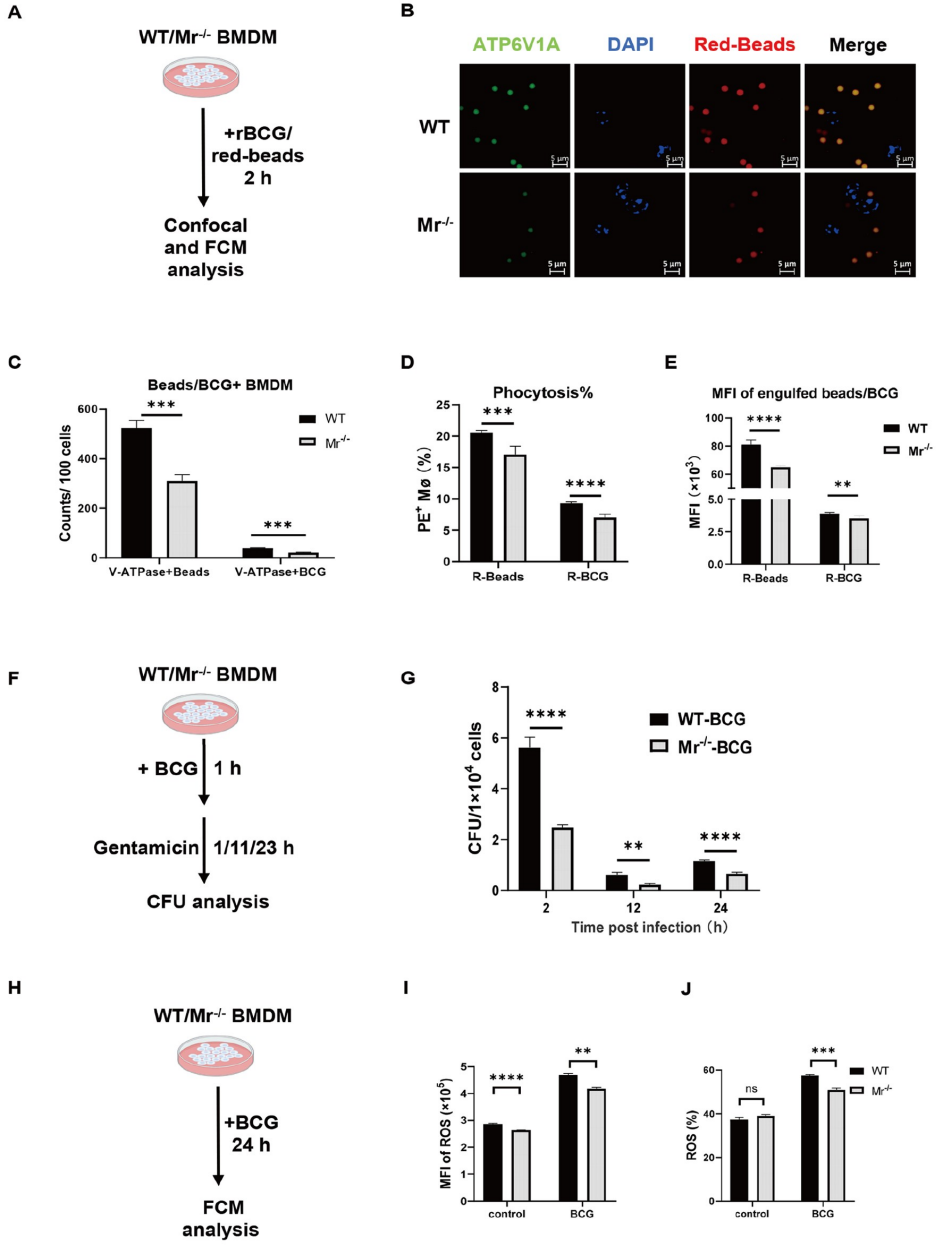
Figure 2 MR deficiency impairs phagocytosis of BCG by macrophages *in vitro* WT and Mr‒/‒ BMDMs were incubated with fluorescent microparticles and mCherry-BCG for 2 h. (A‒C) After the cells were washed and fixed, the amount of engulfed microparticles and BCG were measured by confocal microscopy. (A) Experimental scheme. (B) Representative confocal images (scale bar: 5 μm). (C) Pooled data chart. (D,E) Fluorescence microparticles engulfed by the BMDMs and BCG were measured by FCM. (D) Percentages of engulfed BCG+/microparticle+ macrophages. (E) MFIs of engulfed BCG/microparticles in macrophages. (F,G) WT and Mr‒/‒ BMDMs were stimulated with BCG for 1 h. After washing, the cells were cultured for the indicated durations, and the amount of live BCG engulfed by the cells was determined by plate culture. (F) Experimental scheme. (G) CFUs of engulfed BCG in BMDMs. (H‒J) WT and Mr‒/‒ BMDMs were incubated with BCG for 24 h. ROS release by the cells was measured by FCM. Data are presented as the mean±SD (C,G,I and J, n=3; D and E, n=6) and were evaluated by ANOVA followed by the Neuman-Keuls post hoc test (**P<0.001, ***P<0.001, ****P<0.0001, ns: not significant).

Because most BCG vaccines are taken up by *in situ* macrophages rather than dendritic cells (DCs) in the early period after vaccination [ 13, 14], we assessed the effects of the MR of macrophages on phagocytosis. Fluorescent microparticles (red beads) and BCG (mCherry-BCG) were incubated with BMDMs from WT or *Mr* ^‒/‒^ mice, and the microparticles and BCG engulfed by the cells were measured by confocal fluorescence microscopy ( Figure 2A). As expected, after 2 h of incubation, the numbers of both intracellular microparticles ( Figure 2B,C) and BCG ( Figure 2C and Supplementary Figure S1A) were lower in MR-deficient macrophages than in WT macrophages, demonstrating that MR deficiency impaired macrophage phagocytosis. Similarly, flow cytometry (FCM) analysis revealed a significant reduction in both the percentage of engulfing macrophages (phagocytosis) and the mean fluorescence intensity (MFI) of engulfed BCG and fluorescent microparticles in *Mr* ^‒/‒^ macrophages ( Figure 2D,E and Supplementary Figure S1B).

The engulfed bacteria are lysed within the increased acidic environment of macrophage phagosomes. Therefore, we used a lysosensor probe (PDMPO) to visualize the acidity of phagosomes in macrophages upon stimulation with BCG. Phagosomes in MR-deficient macrophages showed much weaker acidic staining (green) than those in the WT control group, indicating that phagocytosis by MR might facilitate acidification of phagosomes ( Supplementary Figure S1C). Moreover, a bacterial colony counting assay revealed that MR deficiency led to reduced retention of BCG in macrophages, as evidenced by lower CFU (colony forming unit) counts of the intracellular bacteria in *Mr* ^–/–^ BMDMs after 2 h, 12 h and 24 h of incubation with BCG ( Figure 2F,G). FCM analysis also revealed that a greater level of ROS was produced by WT BMDMs than by *Mr* ^‒/‒^ BMDMs ( Figure 2H‒J and Supplementary Figure S1D). The above data strongly suggested that MR deficiency impairs the phagocytosis of BCG by macrophages.

### MR deficiency impairs BCG antigen presentation by macrophages *in vitro*

**Figure 3:**
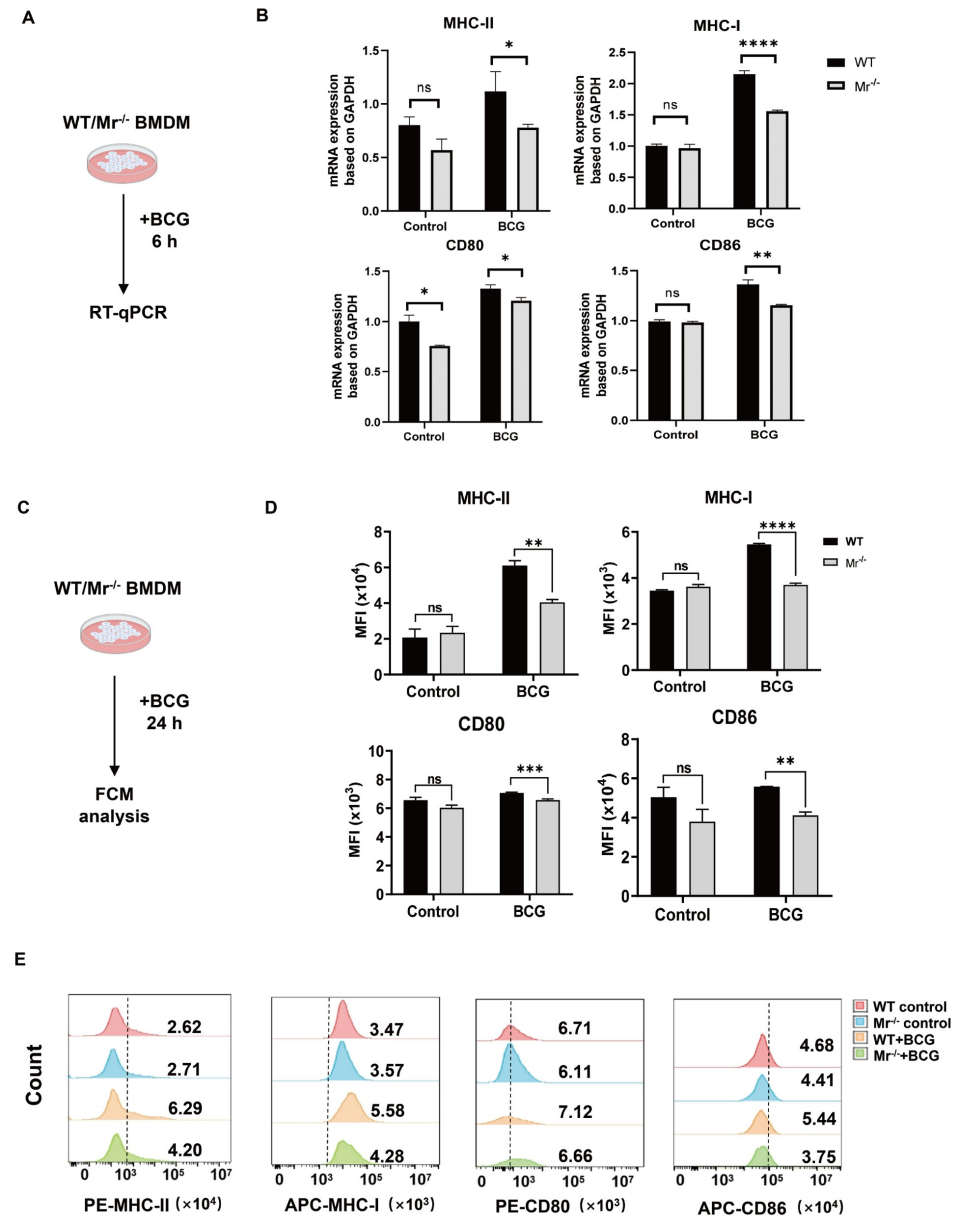
Figure 3 MR deficiency impairs BCG antigen presentation by macrophages *in vitro* (A,B) WT and Mr‒/‒ BMDMs were incubated with BCG for 6 h. The mRNA expression levels of MHC-I/II, CD80 and CD86 were determined by RT-qPCR. (A) Experimental scheme. (B) The mRNA expression levels of MHC-I/II, CD80 and CD86. (C‒E). WT and Mr‒/‒ BMDMs were incubated with BCG for 24 h. The expression levels of the surface proteins MHC-I/II, CD80 and CD86 were determined by FCM. (C) Experimental scheme. (D) Pooled data of the MFIs of MHC-I/II, CD80 and CD86 in macrophages. (E) Representative FCM histograms. Data are presented as the mean±SD (B and D, n=3) and were evaluated by ANOVA followed by the Neuman-Keuls post hoc test (*P<0.05, **P<0.001, ***P<0.001, ****P<0.0001, ns: not significant).

Then, we investigated whether impaired phagocytosis in *Mr* ^‒/‒^ macrophages affects their antigen-presenting capability in the context of BCG vaccination. BMDMs from WT and *Mr* ^‒/‒^ mice were incubated with BCG *in vitro*, and the mRNA expressions of antigen-presenting-related molecules on macrophages were measured by RT-qPCR ( Figure 3A). MR deficiency resulted in a decrease in the mRNA expressions of co-stimulatory molecules (CD80, CD86, and MHC-I/II) in BCG-stimulated BMDMs ( Figure 3B). We also employed FCM analysis to measure the surface protein levels of the above molecules ( Figure 3C). FCM analysis revealed that both the MFIs and percentages of CD80, CD86 and MHC-I/II on the cell surface were lower in *Mr* ^‒/‒^ BMDMs than in WT BMDMs ( Figure 3D,E and Supplementary Figure S2). Here, reductions in the levels of CD80, CD86 and MHC-I/II on the cell surface of BCG-stimulated *Mr* ^‒/‒^ macrophages might contribute to decreasing the T cell response to the vaccine. Taken together, our results clearly suggested that MR deficiency impairs BCG antigen presentation by macrophages *in vitro*.

### MR deficiency impairs BCG antigen presentation by macrophages *in vivo*

**Figure 4:**
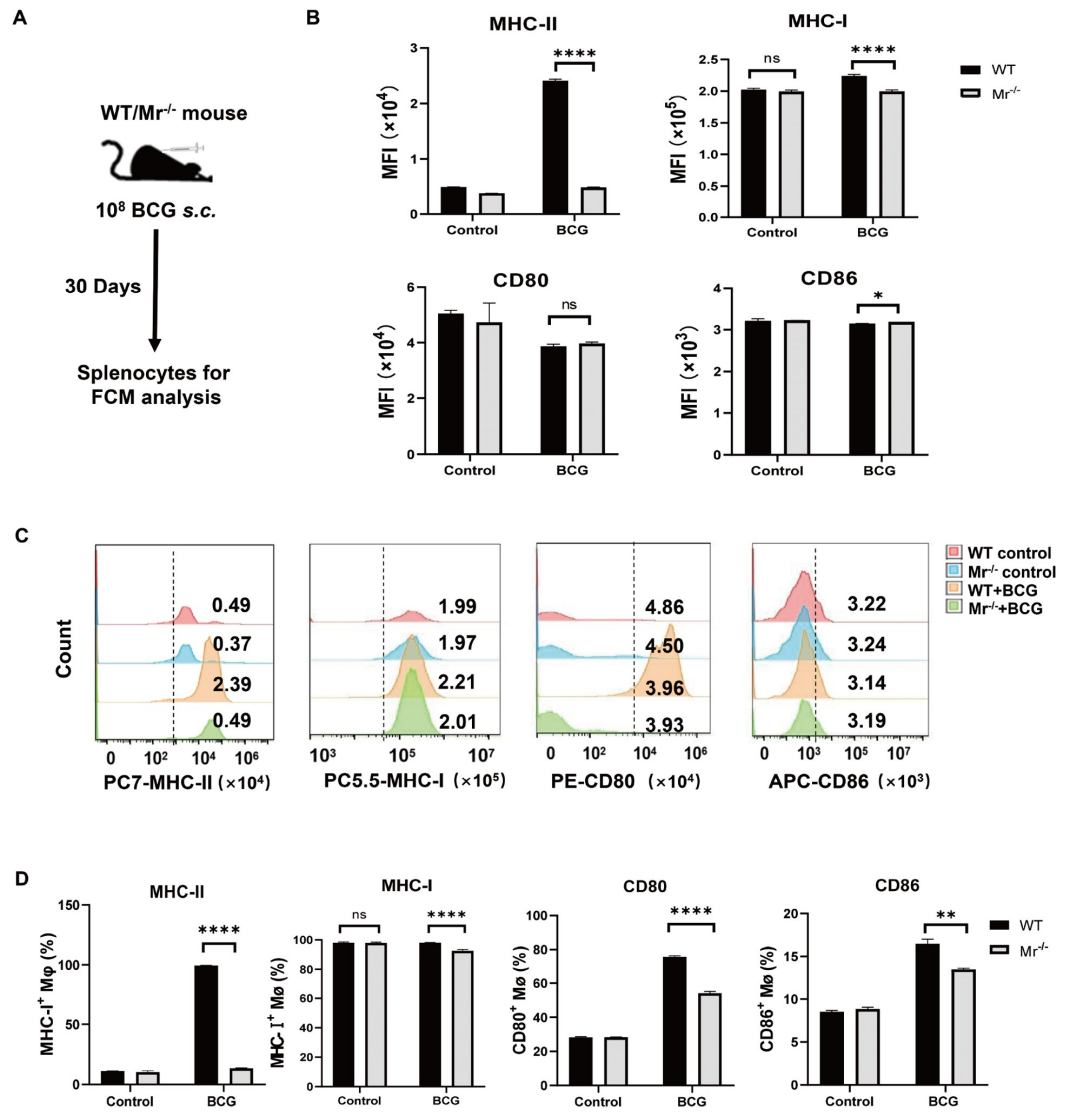
Figure 4 MR deficiency impairs BCG antigen presentation by macrophages *in vivo* Mr‒/‒ and WT mice were vaccinated with BCG. The expression levels of the surface proteins MHC-I/II, CD80 and CD86 on splenic macrophages were determined by FCM on day 30 post vaccination. (A) Experimental scheme. (B) MFIs of the surface proteins MHC-I/II, CD80 and CD86 on splenic macrophages. (C) Representative FCM histograms. (D) Percentages of MHC-I+, MHC-II+, CD80+ and CD86+ macrophages. Data are presented as the mean±SD (B and C, n=6) and were evaluated by ANOVA followed by the Neuman-Keuls post hoc test (*P<0.05, **P<0.001, ****P<0.0001, ns: not significant).

Next, we assessed whether MR affects the antigen-presenting capability of macrophages in a murine model of BCG vaccination. Both WT and *Mr* ^‒/‒^ mice were vaccinated with BCG, and the protein levels of antigen-presenting-related molecules on macrophages were measured on day 30 after vaccination ( Figure 4A). Consistent with our *in vitro* results, *Mr* ^‒/‒^ mice showed much lower MFIs and percentages of MHC-I/II ^+^, CD80 ^+^ and CD86 ^+^ macrophages than did WT mice after vaccination ( Figure 4B‒D). In particular, the expression of macrophage MHC-II in BCG-vaccinated *Mr* ^–/–^ mice was sharply reduced by 90% compared to that in WT mice (13.6% vs 99.3%, *Mr* ^‒/‒^ vs WT; Figure 4C,D). Our findings strongly demonstrated that MR deficiency impairs BCG antigen presentation by macrophages in BCG-vaccinated mice.

### MR deficiency impairs the BCG-induced p-JAK1-p-STAT1-MHC-II pathway in macrophages

**Figure 5:**
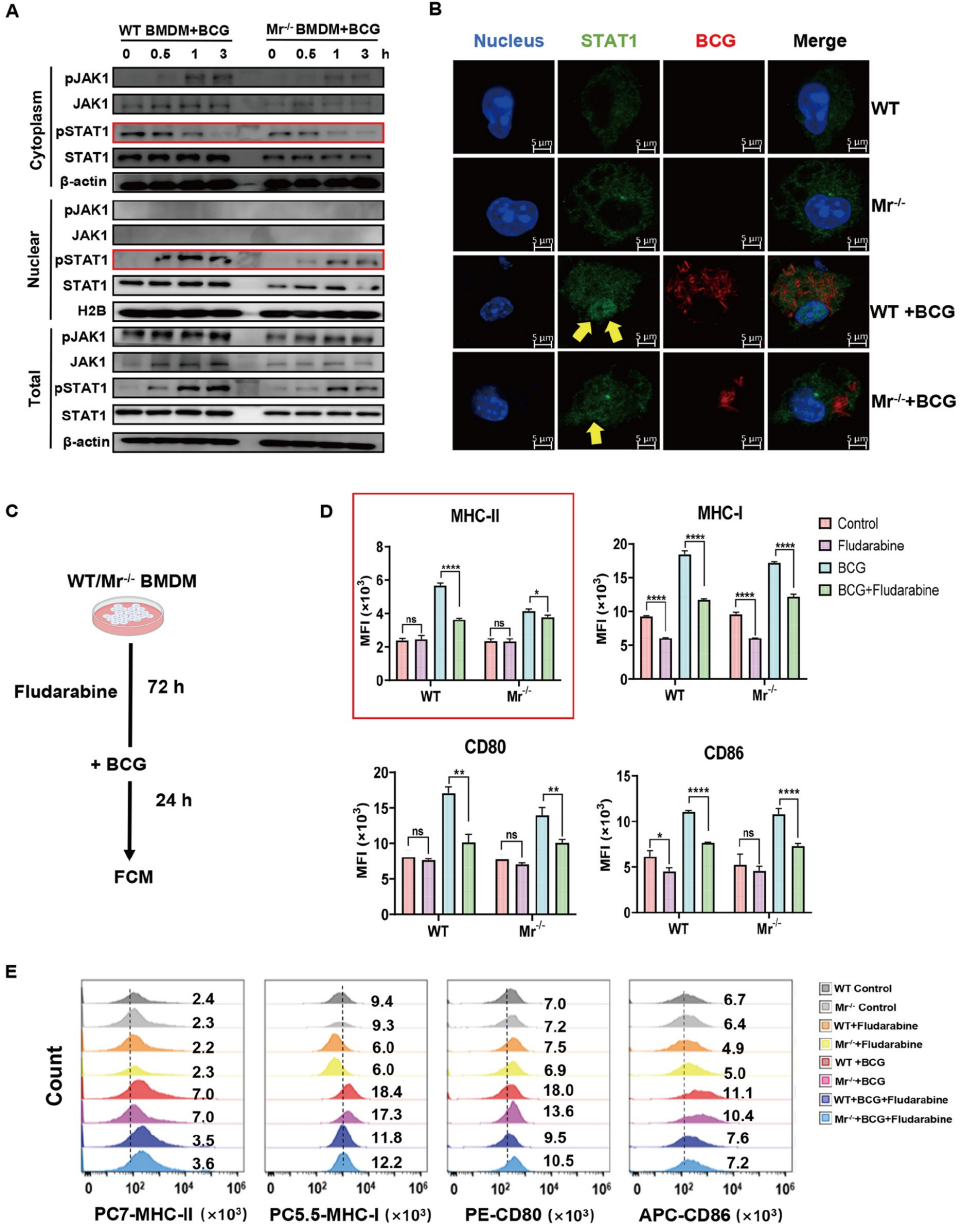
Figure 5 MR deficiency impairs the BCG-induced p-JAK1-p-STAT1-MHC-II pathway in macrophages (A) WT and Mr‒/‒ BMDMs were incubated with BCG for the indicated durations. The nuclear, cytoplasmic and total expression levels of pJAK1, JAK1, pSTAT1 and STAT1 in the cells were determined by immunoblotting analysis. (B) WT and Mr‒/‒ BMDMs were incubated with BCG for 1 h. After the removal of extracellular bacteria by washing, the cells were further cultured for 2 h, and nuclear STAT1 was measured by confocal microscopy (scale bar: 5 μm). WT and Mr‒/‒ BMDMs were treated with the STAT1 inhibitor fludarabine prior to incubation with BCG. The expression levels of the surface proteins MHC-I/II, CD80 and CD86 were analyzed by FCM. (C) Experimental scheme. (D) Pooled MFI data for MHC-I/II, CD80 and CD86 in BMDMs. (E) Representative FCM histogram. Data are presented as the mean±SD (n=3) and were evaluated by ANOVA followed by the Neuman-Keuls post hoc test (*P<0.05, **P<0.001, ****P<0.0001, ns: not significant).

Because the JAK/STAT pathway, a rapid membrane-to-nucleus signaling pathway, induces the expressions of various critical mediators during infection and is essential for the immunomodulation of innate and adaptive immunity [ 15, 16], we investigated this pathway in both WT and *Mr* ^–/–^ BMDMs upon BCG stimulation. BMDMs from *Mr* ^‒/‒^ and WT mice were incubated with BCG for 0.5, 1 or 3 h, and the phosphorylation of JAK1 and STAT1 was determined by immunoblotting analysis ( Figure 5A). A greater reduction in p-JAK1 in the cytoplasm and p-STAT1 in both the cytoplasm and nucleus was found in *Mr* ^‒/‒^ BMDMs than in WT BMDMs after BCG stimulation ( Figure 5A). Reduced localization of STAT1 in the nucleus was also observed in *Mr* ^‒/‒^ BMDMs subjected to BCG stimulation compared with WT BMDMs, as shown by confocal microscopy analysis ( Figure 5B, red arrow,). In line with our above findings, *Mr* ^‒/‒^ BMDMs also showed a reduced ability to engulf mCherry-BCG ( Figure 5B).

To investigate the role of STAT1 phosphorylation in the MR-mediated antigen presentation pathway, we measured the protein levels of CD80, CD86 and MHC-I/II in both *Mr* ^‒/‒^ and WT BMDMs subjected to treatment with the STAT1 inhibitor fludarabine by FCM ( Figure 5C‒E and Supplementary Figure S2). Fludarabine treatment markedly reduced the MHC-II protein level in BCG-stimulated WT BMDMs ( Figure 5D,E; BCG vs BCG+fludarabine, *P*<0.0001), while fludarabine weakly reduced the MHC-II level in *Mr* ^‒/‒^ BMDMs ( Figure 5D,E; BCG vs BCG+fludarabine, *P*<0.05). These results suggest that macrophages may utilize the MR-dependent p-JAK1-p-STAT1 pathway to enhance MHC-II expression and contribute to antigen presentation. However, there were no significant differences in CD80, CD86 or MHC-I levels in the presence of fludarabine between *Mr* ^‒/‒^ and WT BMDMs ( Figure 5D,E). These results suggest that macrophage MR-triggered MHC-II expression is dependent on JAK1-STAT1 signaling, but MR-mediated modulation of CD80, CD86 and MHC-I expressions is not dependent on the STAT1 pathway. Together, our results demonstrated that macrophage MR-triggered MHC-II expression is dependent on JAK1-STAT1 signaling in response to BCG vaccination.

### MR deficiency impairs IL-12 production by macrophages in a STAT1-dependent manner

**Figure 6:**
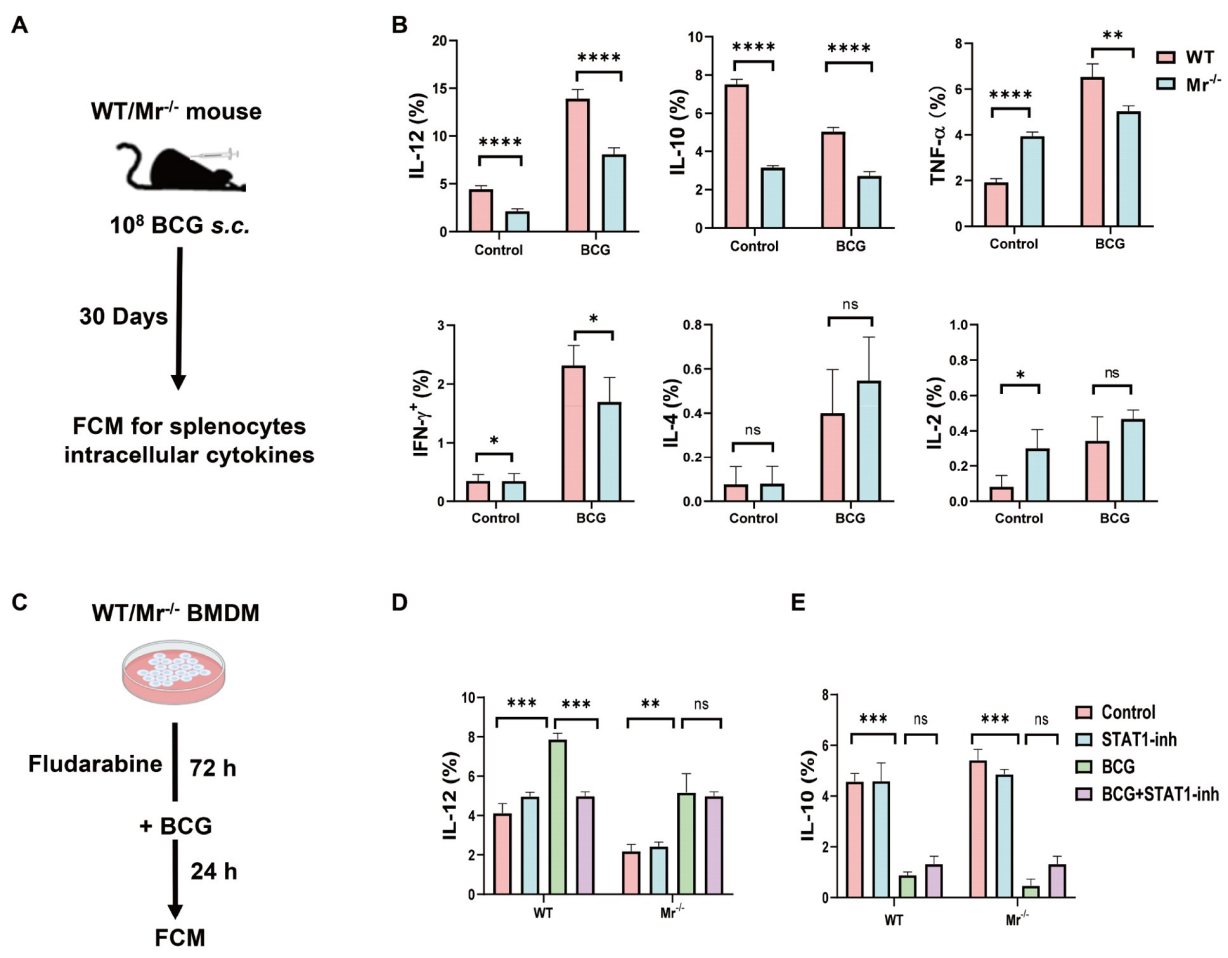
Figure 6 MR deficiency impairs IL-12 production by macrophages in a STAT1-dependent manner Mr‒/‒ and WT mice were vaccinated with BCG. On day 30 post vaccination, the levels of IL-12, IL-10, TNF-α, IFN-γ, IL-4 and IL-2 produced by splenic macrophages were measured by FCM. (A) Experimental scheme. (B) The percentages of IL-12+, IL-10+, TNF-α+, IFN-γ+, IL-4+ and IL-2+ macrophages. BMDMs from Mr‒/‒ and WT mice were treated with the STAT1 inhibitor fludarabine for 72 h and then incubated with BCG for 24 h. The production of IL-12 and IL-10 by the cells was determined by FCM. (C) Experimental scheme. (D,E) Percentages of IL-12+ (D) or IL-10+ (E) macrophages. Data are presented as the mean±SD (B, D and E, n=3) and were evaluated by ANOVA followed by the Neuman-Keuls post hoc test (*P<0.05, **P<0.001, ***P<0.001, ****P<0.0001, ns: not significant).

Next, we assessed the effects of MR on cytokine production by macrophages. WT and *Mr* ^‒/‒^ mice were vaccinated with BCG, and cytokine production by splenic macrophages was determined on day 30 after vaccination. Cytokines, including IL-2, IL-4, IL-12, IFN-γ and TNF-α, are considered important enhancers that promote antigen presentation, T cell activation and antibody production [17], while IL-10 is a crucial regulatory cytokine for immune suppression [18]. Therefore, we detected the production of the above cytokines in BCG-vaccinated mice. As shown in Figure 6A,B and Supplementary Figure S3A, the macrophages from BCG-vaccinated *Mr* ^‒/‒^ mice exhibited lower levels of IL-12, IL-10, TNF-α and IFN-γ production than their counterparts from WT mice, especially for IL-12 ^+^ F4/80 ^+^ macrophages ( Figure 6B; 8.1% vs 14.0%) and IL-10 ^+^ F4/80 ^+^ macrophages ( Figure 6B; 2.7% vs 5.1%). However, the production of IL-4 and IL-2 by macrophages did not differ between *Mr* ^‒/‒^ and WT mice ( Figure 6A,B and Supplementary Figure S3A).

To further evaluate the roles of STAT1 in macrophage MR signaling, we treated WT and *Mr* ^‒/‒^ BMDMs with BCG for 72 h in the presence or absence of a STAT1 inhibitor, and IL-12 and IL-10 production by the BMDMs was measured ( Figure 6C‒E and Supplementary Figure S3B). FCM analysis revealed that the STAT1 inhibitor fludarabine dramatically decreased IL-12 production in BCG-stimulated WT BMDMs but not in *Mr* ^‒/‒^ BMDMs ( Figure 6D,E and Supplementary Figure S3B). These results clearly demonstrate that elevated IL-12 production by BCG-stimulated macrophages is dependent on the MR-STAT1 pathway. The STAT1 inhibitor fludarabine did not alter IL-10 production in either WT or *Mr* ^‒/‒^ BMDMs subjected to BCG treatment, suggesting that STAT1 did not regulate IL-10 production ( Figure 6D,E and Supplementary Figure S3B). Our results demonstrated that MR deficiency impairs IL-12 production by macrophages in a STAT1-dependent manner.

### MR deficiency in macrophages impairs T cell proliferation and activation in an IL-12-dependent manner

**Figure 7:**
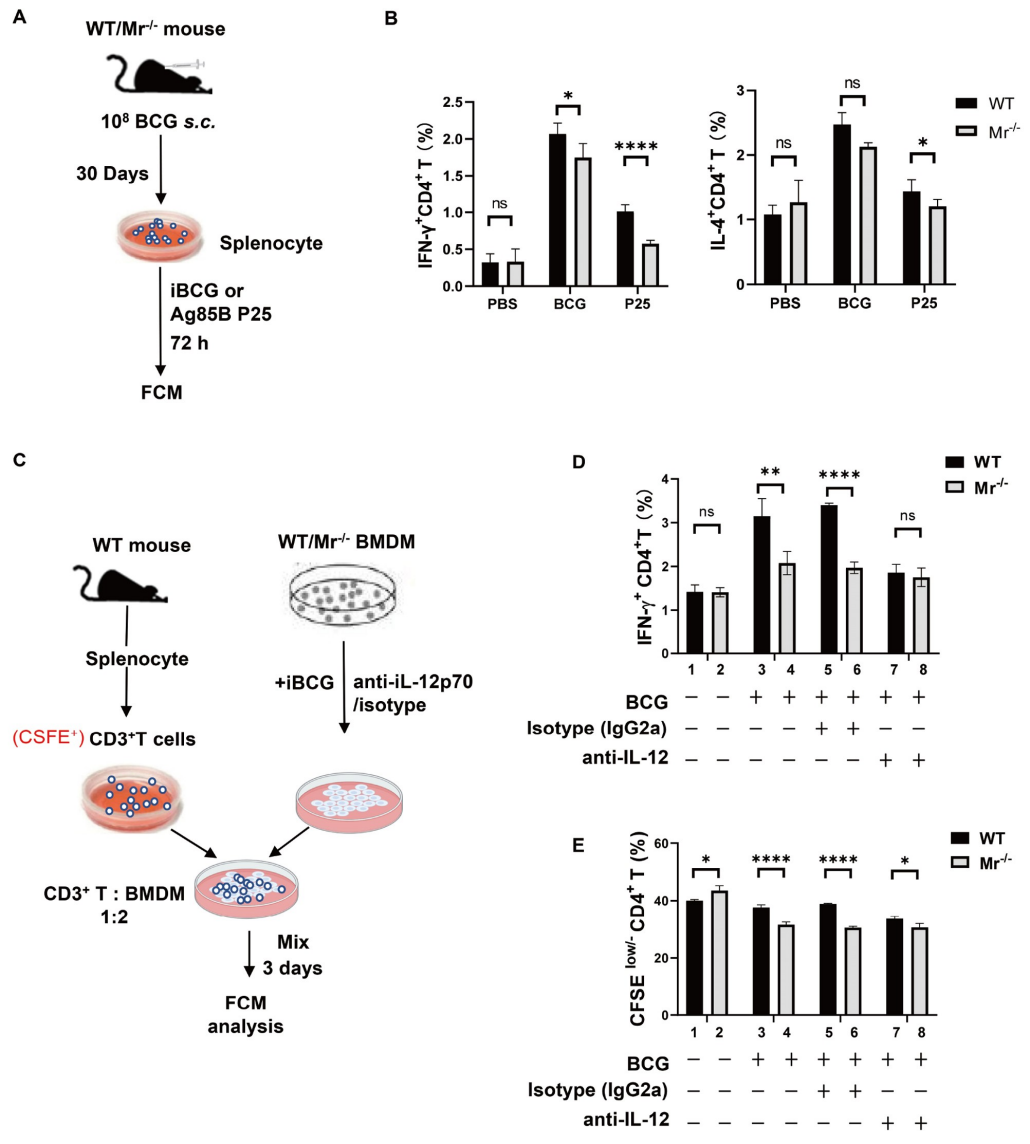
Figure 7 MR deficiency in macrophages impairs T cell proliferation and activation in an IL-12-dependent manner WT and Mr‒/‒ mice were vaccinated with BCG. On day 30 post vaccination, the splenocytes from the mice were restimulated with iBCG or Ag85B P25 in vitro, and the levels of IFN-γ and IL-4 produced by splenic CD4+ T cells were measured by FCM. (A) Experimental scheme. (B) The percentages of IFN-γ+ and IL-4+ CD4+ T cells. MACS-purified splenic CD3+ T cells were incubated with BCG antigen-loaded WT (or Mr‒/‒) BMDMs in the presence of an anti-IL-12p70 antibody for 3 days. IFN-γ production by CD4+ T cells and CD4+ T cell proliferation were measured by FCM. (C) Experimental scheme. (D) The percentage of IFN-γ+ CD4+ T cells. (E) The percentage of CFSElow/‒ CD4+ T cells. Data are presented as the mean±SD (B, n=6; D, n=3) and were evaluated by ANOVA followed by the Neuman-Keuls post hoc test (*P<0.05, **P<0.001, ****P<0.0001, ns: not significant).

Because the MHC-II antigen presentation pathway mainly triggers CD4 ^+^ T cell activation, we assessed the effects of MR-mediated antigen presentation on CD4 ^+^ T cell activation and differentiation in BCG-vaccinated mice. On day 30 post vaccination, the splenocytes from *Mr* ^‒/‒^ and WT mice were restimulated with inactive BCG (iBCG) or the mycobacterial antigen Ag85B peptide P25 [19] *in vitro* ( Figure 7A), and antigen-specific IFN-γ-producing CD4 ^+^ cells and IL-4-producing CD4 ^+^ cells were measured. As shown in Figure 7B and Supplementary Figure S4A, MR deficiency led to much lower percentages of IFN-γ ^+^CD4 ^+^ T cells regardless of whether these cells were restimulated with iBCG or P25 *in vitro*, demonstrating that MR deficiency impaired the development of the BCG antigen-specific CD4 ^+^ Th1-mediated immune response. However, no or a low reduction (less than 3%) in the percentage of IL-4 ^+^CD4 ^+^ Th2 cells was observed in the iBCG- or P25-restimulated *Mr* ^‒/‒^ groups ( Figure 7B and Supplementary Figure S4A), demonstrating that MR impacts the immune response mainly through IFN-γ ^+^CD4 ^+^ Th1 cells.

To further evaluate the role of IL-12 produced by macrophages during T cell activation, we treated WT and *Mr* ^‒/‒^ BMDMs with iBCG *in vitro*, and these antigen-loaded BMDMs were used to activate the sorted T cells from WT mice in the presence of anti-IL-12 or an isotype control ( Figure 7C‒E and Supplementary Figure S4B,C). In line with the *in vivo* results shown in Figure 7B, a much lower level of IFN-γ production by CD4 ^+^ T cells was observed when these T cells were incubated with *Mr* ^‒/‒^ BMDMs in mixed lymphocyte reaction (MLR) analysis ( Figure 7D). More importantly, blocking IL-12 signaling diminished the difference in IFN-γ production between the WT and *Mr* ^‒/‒^ groups ( Figure 7D; 7th vs 8th columns). Similarly, the proliferation of T cells was suppressed in the *Mr* ^‒/‒^ group compared with that in the WT group, while anti-IL-12 treatment sharply decreased the difference in the percentage of proliferated cells between the WT and *Mr* ^‒/‒^ groups ( Figure 7E; 7th vs 8th column). Our results suggested that MR deficiency in macrophages impairs T cell proliferation and activation in an IL-12-dependent manner.

### MR deficiency impairs the antimycobacterial immune response after BCG vaccination

**Figure 8:**
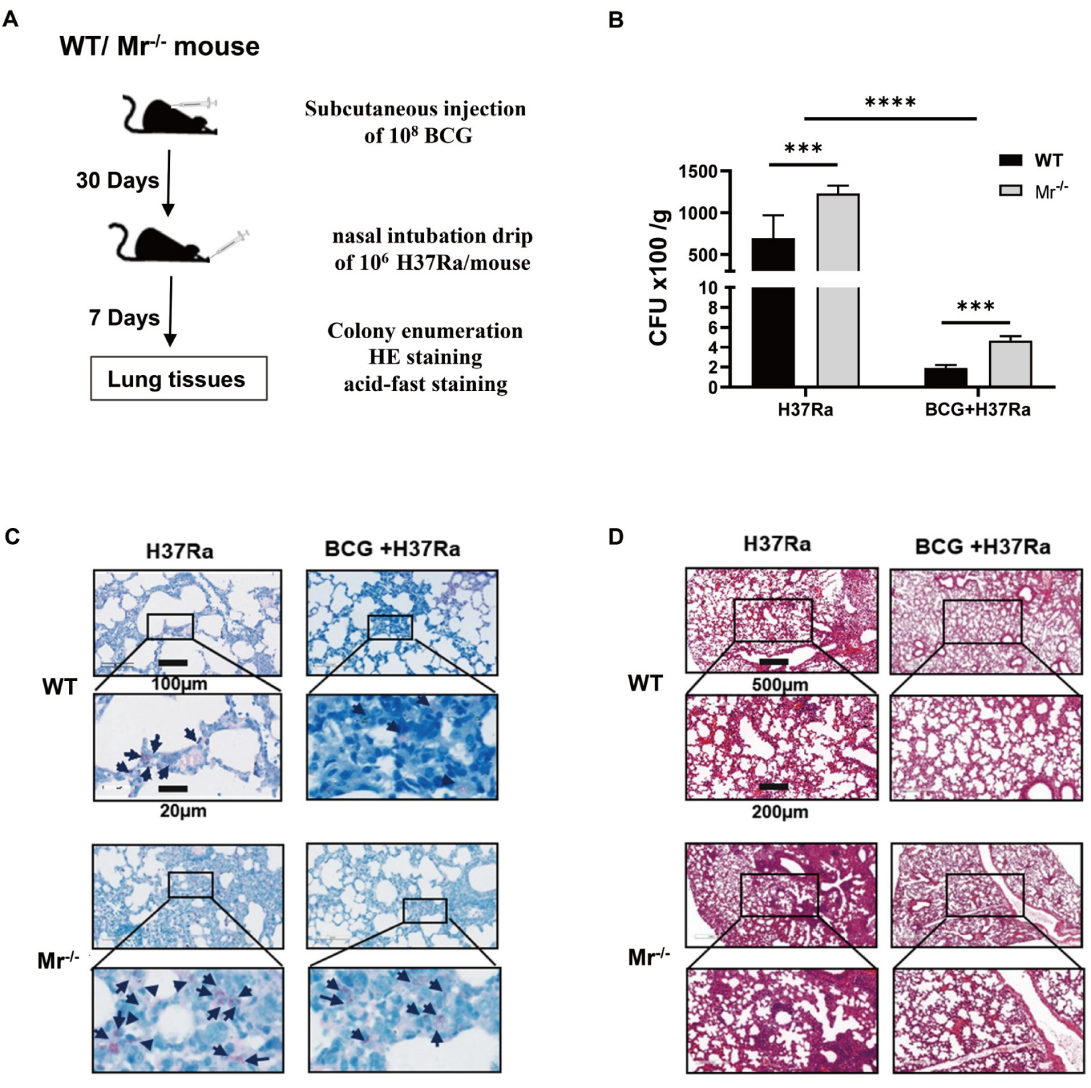
Figure 8 MR deficiency impairs the antimycobacterial immune response after BCG vaccination Mr‒/‒ and WT mice were vaccinated with BCG on day 0. The mice were infected with M.tb H37Ra. (A) Experimental scheme. (B) M.tb H37Ra loads in lung tissues were measured by plate culture on day 7 post infection. (C) M.tb H37Ra loads in the lung tissues were analyzed with Ziehl-Neelsen acid-fast staining. The arrows represent the bacteria. (D) Lung tissues were stained with hematoxylin and eosin (H&E). The levels of IFN-γ and IL-10 produced by CD4+ T cells were determined by FCM. Data are presented as the mean±SD (B, n=3) and were evaluated by ANOVA followed by the Neuman-Keuls post hoc test (***P<0.001).

The above findings indicate that MR-mediated BCG antigen presentation by macrophages may contribute to T cell proliferation. Therefore, we investigated whether macrophages utilize MR to promote the immune response against *M*. *tb* infection after BCG vaccination in mice. WT and *Mr* ^‒/‒^ mice were vaccinated with BCG on day 0, and the mice were infected with *M*. *tb* H37Ra on day 30. After 7 days of infection, the bacterial burden and pathological changes in the lungs were measured ( Figure 8A). As shown in Figure 8B, compared with the unvaccinated and H37Ra infection groups, BCG vaccination decreased the mycobacterial H37Ra CFUs in the lungs of both WT and *Mr* ^‒/‒^ mice. However, MR deficiency resulted in an increase in the bacterial burden in both the vaccinated and unvaccinated groups compared with that in the corresponding WT group ( Figure 8B). In line with the results of the CFU analysis, acid-fast staining analysis further confirmed that BCG-vaccinated *Mr* ^‒/‒^ mice exhibited greater mycobacterial colonization than BCG-vaccinated WT mice ( Figure 8C). Hematoxylin and eosin (H&E) staining revealed that MR deficiency also aggravated lung lesions in *M*. *tb*-infected *Mr* ^‒/‒^ mice ( Figure 8D). We observed larger areas of alveolar wall destruction and inflammatory cell infiltration in *M*. *tb*-infected *Mr* ^‒/‒^ mice ( Figure 8). These results demonstrated that MR deficiency impairs the antimycobacterial immune response after BCG vaccination.

## Discussion

BCG, as the only available vaccine for TB, has been used for more than 60 years. BCG provides limited protection against adult pulmonary TB; the protection in adults varies from zero to 80%. To date, the precise reasons underlying the inability of BCG vaccination to induce a robust antimycobacterial immune response in adults remain elusive.

Previous research has revealed a significant role for MR in the phagocytic activity of human alveolar macrophages against *M*. *tb*. Macrophages utilize MR for the clearance of a variety of pathogens, including *Candida albicans* [20], *Leishmania* [21], *Klebsiella pneumoniae* [22] and *M*. *tb* [9]. In addition to binding to and internalizing a variety of pathogens, MR may also play a central role in enhancing the uptake and processing of glycoconjugates from pathogens for presentation to T cells [ 23, 24]. MR activation in tumor-associated macrophages enhances adaptive and innate antitumor immune responses [25]. MR reportedly binds to the lipoprotein LpqH and mannose-capped lipoarabinomannan (ManLAM) on the surface of BCG/ *M*. *tb* [ 26– 28]. We and other groups have previously reported that MR binds to mycobacterial ManLAM and that this binding significantly influences the fate of engulfed *M*. *tb* in macrophages as well as the activation and augmentation of both innate and adaptive immune responses [ 9, 26, 29– 31]. However, the roles of the macrophage MR in the BCG vaccine-induced innate and adaptive immune responses against *M*. *tb* are unknown.

Here, we demonstrated that MR deficiency impairs antigen presentation by BCG-treated macrophages. We found that macrophage MRs facilitated phagocytosis and ROS production, as well as the upregulation of MHC-II and IL-12 production and BCG antigen presentation in macrophages. According to our results, MR is required for the development of an antimycobacterial immune response during BCG vaccination, and this requirement might be due to the highly mannosylated lipids and proteins of PAMPs on the surface of the BCG bacilli, which favors the enhancement of bacillus engulfment by macrophages via MR. Our results revealed that host MR is necessary in the BCG-mediated immune response. It has been reported that the serum soluble macrophage MR and sCD206 are increased in pulmonary TB (PTB) patients and are associated with poor prognosis [32]. Based on the present study and others’ reports, we speculate that the decreased immune protection of BCG against *M*. *tb* might be related to varied MR expression levels in adults and that decreased or varied MR expression might contribute to an inefficient antimycobacterial response in adults after BCG vaccination. Although we revealed the role of the macrophage MR in the antimycobacterial response, the potential role of MR in other immune cells, such as dendritic cells, merits future studies.

Recent data showed that the JAK-STAT1 transcriptional signature is characteristic of an “M1” proinflammatory monocyte and macrophage phenotype [33]. *STAT1* plays an important role in immune defense against TB infection and is recognized as one of the key genes related to the interferon signaling pathway. Here, we found that engagement of the macrophage MR triggers JAK-STAT1-MHC-II signaling, which promotes antigen presentation via upregulation of MHC-II. The STAT1 inhibitor fludarabine markedly reduced the level of MHC-II in BCG-stimulated macrophages but had a slight effect on that in the *Mr* ^‒/‒^ group, suggesting that enhanced MHC-II expression in macrophages contributes to antigen presentation in a STAT1-dependent manner. According to our results, JAK-STAT1-MHC-II signaling triggered by MR in macrophages promotes antigen presentation and consequently more effectively stimulates CD4 ^+^ T cells. MR lacks an intracellular signaling motif, but the receptor is associated with TLR2/4 and the FcRγ chain to enhance the host immune response [ 9, 34, 35]. The binding of MR to its ligands has also been shown to trigger the activation of the Rac/Pak/Cdc-42 signaling cascade and PPAR-γ during *M*. *tb* infection [ 9, 35, 36]. We speculate that MR might interact with TLR2, TLR4 or FcR, leading to the activation of JAK-STAT1-MHC II signaling and the enhancement of antigen presentation during BCG vaccination.

IL-12 has been reported to selectively participate in T cell activation and memory T cell development [37]. In the present study, we also found that MR increased IL-12 production by BCG-treated macrophages. The STAT1 inhibitor fludarabine dramatically decreased IL-12 production in BCG-stimulated WT BMDMS but not in the *Mr* ^‒/‒^ group, suggesting that elevated IL-12 production by BCG-stimulated macrophages is dependent on the MR-STAT1 pathway. We also discovered that the MR of macrophages promotes IFN-γ production, CD4 ^+^ T cell proliferation, and the specific antimycobacterial immune response of CD4 ^+^ T cells after BCG vaccination. Therefore, we propose that macrophages exploit MR to engulf the BCG vaccine, upregulating the expression of MHC-II and the production of IL-12, contributing to the development of T cell activation and the promotion of the anti- *M*. *tb* immune response.

To our knowledge, this is the first report demonstrating the roles of MR macrophages in the BCG-induced immune response against *M*. *tb*. Our data showed that macrophages exploit MR to trigger the JAK-STAT1 pathway to upregulate MHC-II expression and IL-12 production, which in turn enhances T cell proliferation and IFN-γ production in the immune response to *M*. *tb* infection. Our results will provide insight for elucidating the mechanism of MR for BCG vaccination and will aid in the development of new strategies against tuberculosis.

## Supporting information

24244Supplementary

## Supplementary Data

Supplementary data is available at *Acta Biochimica et Biophysica Sinica* online.
