# Supplementary material for: Macrophages exploit the mannose receptor and JAK-STAT1-MHC-II pathway to drive antigen presentation and the antimycobacterial immune response after BCG vaccination: MR deficiency impairs BCG immune response
Source: Acta Biochim Biophys Sin (Shanghai). 2024 Jun 18;56(8):1130–44. doi: 10.3724/abbs.2024100 (PMC11399420; doi:10.3724/abbs.2024100)
Supplement: 24244Supplementary [file 24244Supplementary.pdf]

Fig. S1

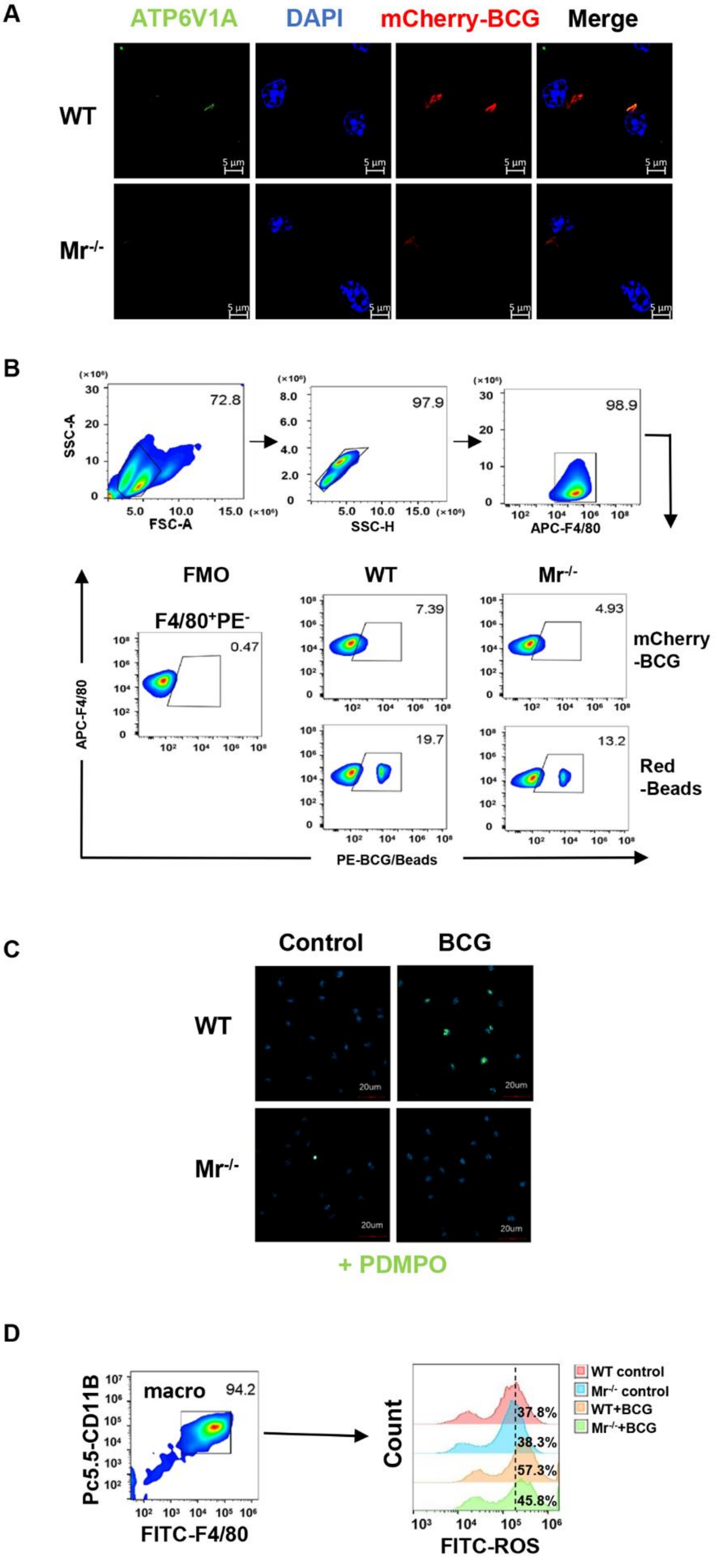

Supplementary Figure S1. MR deficiency impairs phagocytosis of BCG by macrophages and reduces ROS production Wild-type (WT) and *Mr*$^{-/-}$ BMDMs were incubated with fluorescent microparticles and BCG for 2 h. After washing,

engulfed microparticles and BCG were measured by FCM. (A) Representative confocal images (scale bar: 5 μm). (B) The gating strategy and representative FCM plots. (C) The cells were stained with lysosensor probe PDMPO to measure their acidic phagosome activity (scale bar: 20 μm). Representative confocal images. (D) WT and *Mr*$^{-/-}$ BMDMs were stimulated with BCG for 24 h. ROS release by the cells was measured by FCM. Representative FCM plots.

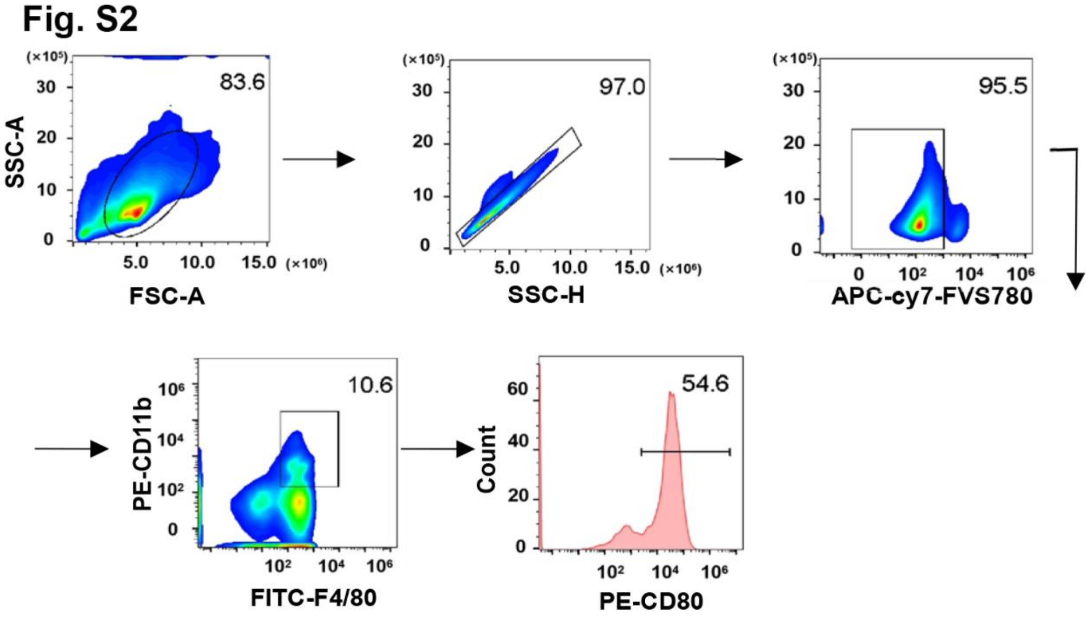

Supplementary Figure S2. FCM gating strategy for detecting the surface antigen presenting-related molecules in macrophages

Fig. S3

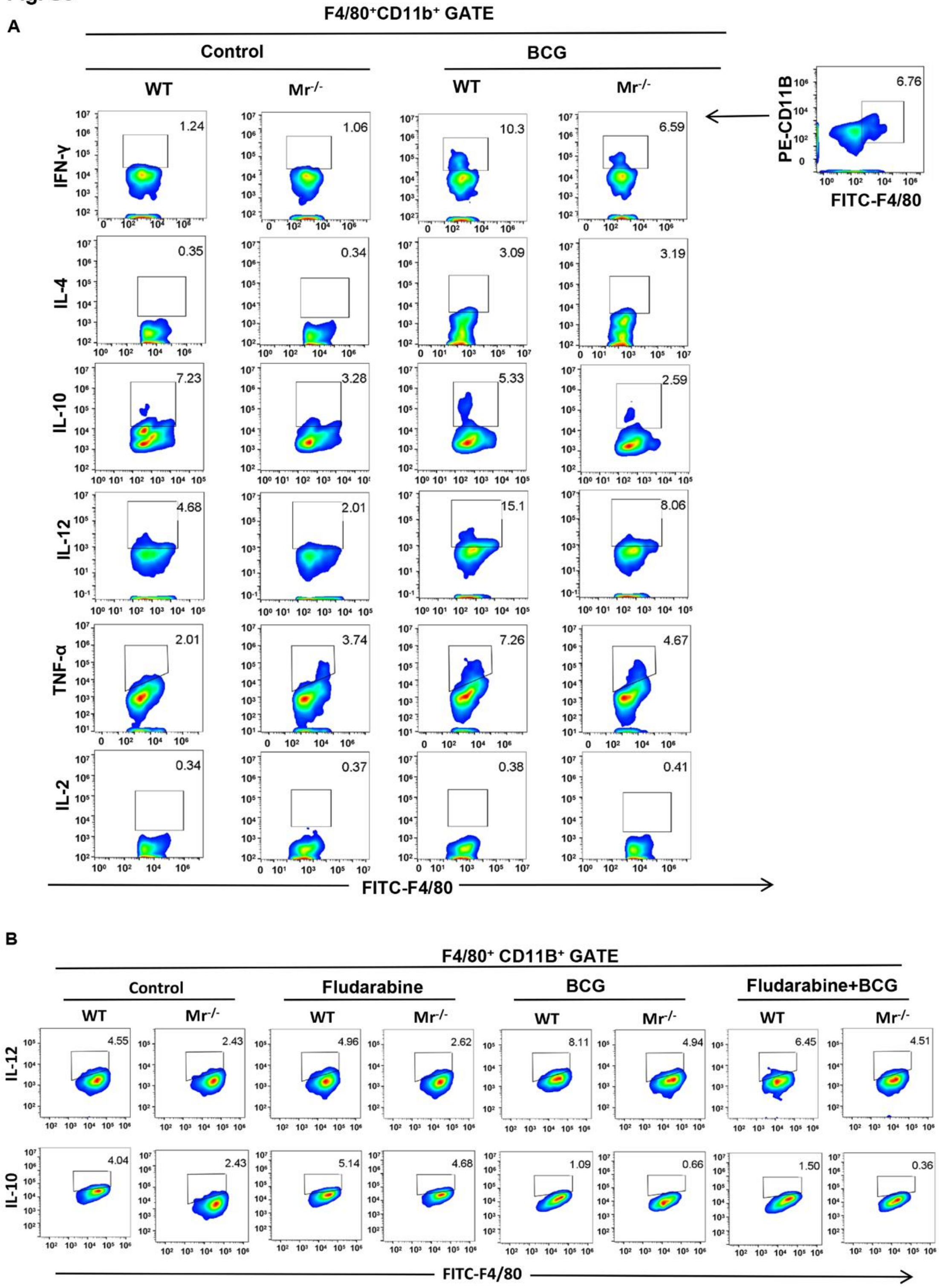

Supplementary Figure S3. MR deficiency impairs IL-12 production of macrophages in a STAT1-dependent manner (A) *Mr*$^{-/-}$ and wild-type (WT) mice were vaccinated with BCG. On Day 30 post the vaccination, the production levels of IL-12, IL-10, TNF-α, IFN-γ, IL-4 and IL-2 by splenic macrophages were measured by FCM. Representative FCM plots. (B) BMDMs from *Mr*$^{-/-}$ and WT mice were treated with STAT1 inhibitor fludarabine for 72 h, and then incubated with BCG for 24 h. The production of IL-12 and IL-10 by the cells was determined by FCM.

Representative FCM plots.

Fig S4

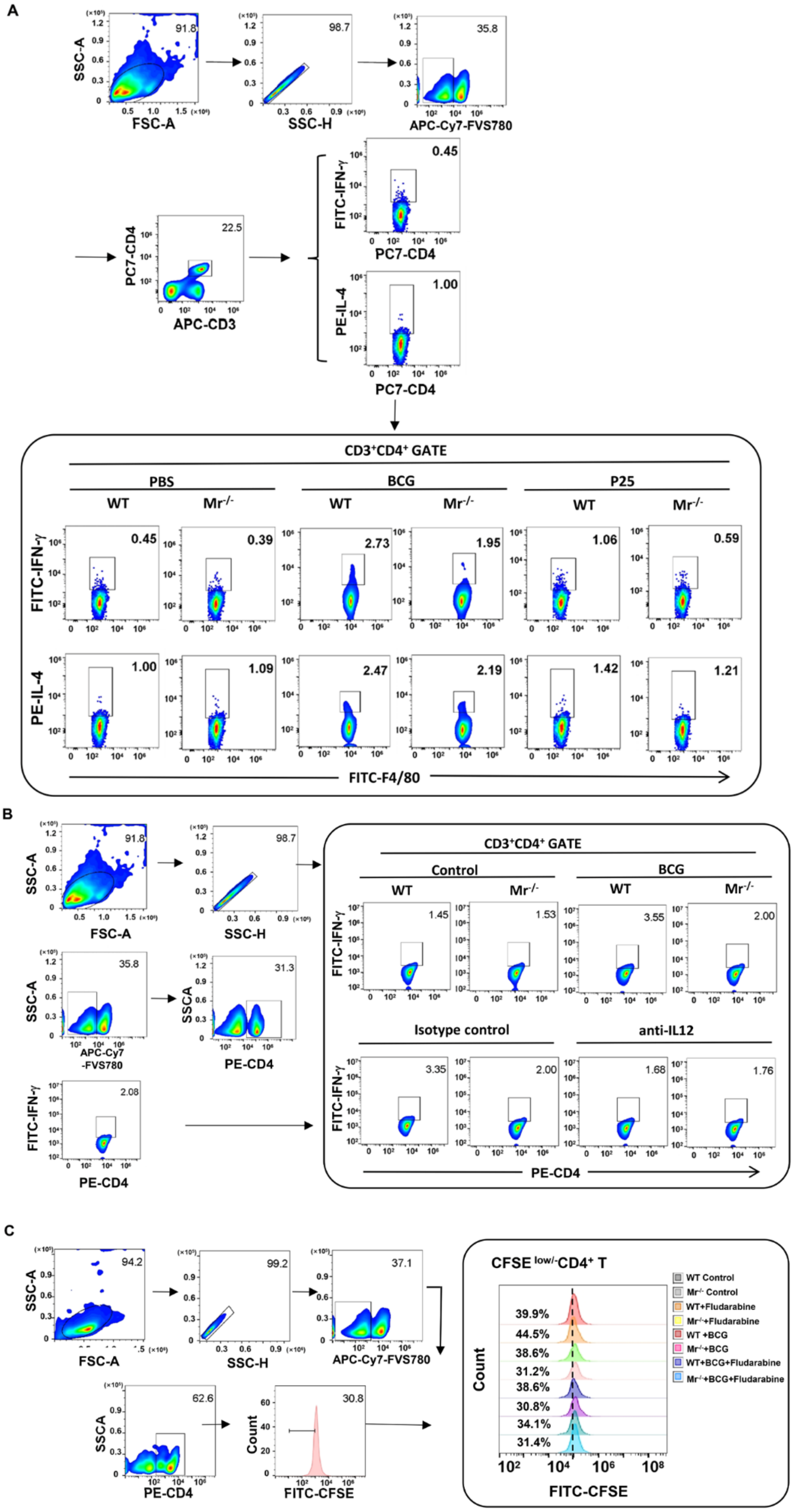

Supplementary Figure S4. MR deficiency of macrophages suppresses IFN-γ

production and proliferation of CD4$^+$ T cells in an IL-12-dependent manner (A) Wild-type (WT) and *Mr*$^{-/-}$ mice were vaccinated with BCG. On day 30 post the vaccination, the splenocytes from the mice were restimulated with iBCG or Ag85B P25 *in vitro* and the production levels of IFN-γ and IL-4 by splenic CD4$^+$ T cells were measured by FCM. The gating strategy and representative FCM plots. (B,C) MACS purified splenic CD3$^+$ T cells were incubated with BCG antigen-loaded WT (or *Mr*$^{-/-}$) BMDMs in the presence of anit-IL-12p70 antibody for 3 days. The IFN-γ production by CD4$^+$ T cells and CD4$^+$ T cell proliferation were measured by FCM. (B) The percentages of IFN-γ$^+$ CD4$^+$ T cells. The gating strategy and representative FCM plots. (C) The percentages of CFSE$^{low/-}$ CD4$^+$ T cells. The gating strategy and representative FCM histograms.

Supplementary Table S1. The sequences of primers used for RT-qPCR

Primer	Sequence (5′→3′)
CD80-F	CTTTCAGACCGGGGCACATA
CD80-R	GAAGCGAGGCTTTGGGAAAC
CD86-F	CTTACGGAAGCACCCACGAT
CD86-R	CGGCAGATATGCAGTCCCAT
MHC-I-F	CTGAGATGGAGTAAGGAGAGTGTGG
MHC-I-R	TTGGAGACAGTGGATGGAGGA
MHC-II-F	GCCGACCACGTAGCCTTCTA
MHC-II-R	TTCTTGCAGTCCACCTTGGGG
